# Nutritional status and dietary pattern of the elderly in Thulamela Municipality of Vhembe District

**DOI:** 10.4102/phcfm.v14i1.3439

**Published:** 2022-11-03

**Authors:** Selekane A. Motadi, Tshifhiwa Khorommbi, Lungile Maluleke, Anzani Mugware, Lindelani Mushaphi

**Affiliations:** 1 Department of Nutrition, Faculty of Health Sciences, University of Venda, Thohoyandou, South Africa

**Keywords:** nutritional status, dietary pattern, elderly, dietary intake, body mass index

## Abstract

**Background:**

Dietary patterns provide comprehensive information about the food consumption habits within a population and how an individual’s dietary pattern may change with age.

**Aim:**

To evaluate the nutritional status and dietary patterns of the elderly in Thulamela municipality of Vhembe district, Limpopo province.

**Setting:**

Study was conducted in Thulamela municipality of Vhembe district, Limpopo province.

**Methods:**

This study included 300 elderly people recruited from Thulamela municipality of Vhembe district, Limpopo province. The municipality was randomly selected, and convenience sampling was used to choose elderly people. Body weight and height were measured using standard techniques. Body mass index (BMI) was determined and classified using BMI categories. Data on dietary patterns and dietary intake were collected using a food frequency questionnaire.

**Results:**

A total of 300 elderly people from villages participated in the study. About 38.6% of elderly people had a primary education, while 28.1% had a secondary education. The prevalence of underweight, overweight and obesity was 2.0%, 34.0% and 17.0%, respectively. Less than half of the elderly people did not meet the reference intake of energy (*p* = 0.023). More than half of the elderly people did not meet the reference intake of protein, iron, zinc, potassium, calcium and vitamins B1, B12 and C. It was found that 13.7% of the elderly skipped breakfast every day. About 19.6% of the elderly ate supper sometimes, while 13.7% did not eat supper.

**Conclusion:**

The findings of the study revealed that most of the participants ate three main meals a day, with a minority skipping either breakfast or dinner or eating in between meals. The prevalence of underweight was low and that of overweight and obesity was high.

**Contribution:**

Poor food choices and physiological changes may reduce the inclusion of food rich in minerals and vitamins in the elderly’s diets and prompt the prevalence of malnutrition.

## Introduction

Ageing refers to irreversible continuous changes such as physiological decline in physical and mental abilities which occur from birth to death, and these changes may adversely affect food choice and dietary intake.^1^ The number of elderly people in sub-Saharan Africa is expected to increase from 36.6 million to 141 million between 2005 and 2050, with a large proportion of them in the rural area.^2^ In South Africa, the number of elderly people aged 60 years has grown from 7.6% in 2022 to 9.1% in 2020.^3^ The number of elderly people in South Africa has increased from 2.8 million in 1996 to 4.1 million in 2011, with Limpopo having 8.7% of the entire population of elderly people.^4^

Appropriate nutrition is vital for elderly people because of the physiological changes that occur as a result of ageing.^5^ Loss of muscle mass, metabolic abnormalities and reduced immune function are the results of functional limitations and poor diet in most elderly people.^6^ Good nutritional status and proper diet during ageing are important for boosting immune system and infection resistance.^7^ Eating varieties of food to meet their nutritional needs during their changing physiological state is very rare.^8^ Past experiences of food during childhood and earlier adulthood may influence attitudes concerning food and meals in later life.^9^ However, dietary patterns are not static and may change as the individual is ageing.

Increasing the macronutrients (such as porridge, bread and meat) and micronutrients (such as fruits and vegetables) from different foods in the diet of elderly people will help to meet their nutritional needs.^10^ The inclusion of five servings of food per day, such as fruits, vegetables and protein, may also contribute to an active immune system. Elderly people are at nutritional risk not only because of physiological changes but also because of inadequate food intake, which can result in macro- and micronutrient deficiencies.^11^ Poor quality of diets is the leading cause of macro- and micronutrient deficiencies in sub-Saharan Africa.^12^

In African countries, several studies reported limited research on the health and well-being of the elderly, and interventional strategies to address the needs of this group are poorly articulated. In elderly people, the leading factor to increased hospital admission, morbidity and higher rates of mortality is malnutrition.^13^ South Africa, like many developing countries, is undergoing nutrition transition, which promotes and exacerbates weight gain in age groups. Although dietary changes caused by this nutrition transition are occurring, undernutrition among the elderly still prevails, especially in rural areas. Factors contributing to noncommunicable diseases during old age are lack of physical activity, excessive weight gain and inadequate dietary intake.

In 2008, the prevalence of malnutrition among elderly people in sub-Saharan Africa varied according to the country, with Cameroon at 6.0%, Ghana at 48.0% and Uganda at 68.0% for women and 32.0% for men.^2^ Overweight and obesity were prevalent in most sub-Saharan African countries, with South Africa and Botswana having the highest number of obese women.^2,14^ However, Limpopo (77.1%) and the Eastern Cape (64.4%) provinces were found to have the highest proportion of elderly people who are poor.^4^ Despite the pension income that the elderly receive in South Africa, household food insecurity has been found to be higher in elderly-headed households as a result of poverty risk and large family size.^14,15^ In Limpopo province, deprived areas are mostly found in the rural areas, where a considerable proportion of the population can be categorised as elderly. Therefore, the aim of the study was to assess the nutritional status and dietary patterns of elderly people in Thulamela municipality of Vhembe district. In order to attain the aim of the study, the following objectives were developed:

- to determine the sociodemographic characteristics of the elderly people
- to assess the nutritional status of the elderly people using weight and height
- to assess the dietary intake and patterns of elderly people using a questionnaire.

## Methods

### Study design

The study design was descriptive. This study described the nutritional status and dietary patterns of elderly people. Information was gathered through a quantitative method, which is a method used to collect data or information that deals with numbers or anything else that can be measured. In quantitative research, data are gathered using formal tools like questionnaires and standardised methods. Data are analysed using statistical methods to increase objectivity.^16^

### Study population and sampling strategy

The study was conducted in 12 rural villages of Thulamela municipality in Vhembe district. Thulamela municipality has an estimated population size of 618 462, of which 2 983 422 (6.2%) are over 65 years of age,^3^ and has 227 villages and 156 594 households. The municipality, which makes up 10.0% of the district’s geographic area, is the smallest of the district’s four municipalities. In terms of population, it is the province’s largest municipality. The word Thulamela, which means ‘the site of giving birth’, is of Karanga origin.^17^ Data were collected during a 6-month period (March 2019 to August 2019). A simple random sampling method was used to select villages from 227 villages in Thulamela municipality. A list of elderly people was obtained from the chief’s kraals, and each elderly person was assigned a number; these numbers were placed in a bowl and mixed thoroughly. The researcher picked one number at a time from the bowl blindfolded until the desired number was reached. Each number represented an elderly person. Slovin’s formula was used to calculate the total sample size. The formula yielded 364 participants, and an addition of 10% was added for attrition. Four hundred elderly people were selected conveniently from villages in Thulamela municipality. Slovin’s formula was again used to calculate the number of elderly people to be interviewed in each village. In each village, 33 elderly people were interviewed, while in some villages 34 elderly people were interviewed until the desired number was reached. The number was reduced to 300 elderly people because of illnesses, some being deceased after signing the consent form, withdrawal from the study and missing values of some variables when data were analysed.

### Data collection

Data were collected by a group of student nutritionists (fieldworkers) from March 2019 to August 2019. The fieldworkers entered all households in the selected village and interviewed elderly people in their local language, which was Tshivenda. A pretested and piloted interviewer-administered questionnaire consisting of anthropometric information, demographic data, dietary patterns and 24-h recall was used as a data collection tool. An adapted quantitative food frequency questionnaire was also used to collect data. The quantitative food frequency questionnaire was adapted from the National Food Consumption Survey (NFCS)^18^ to accommodate the inclusion of indigenous food. To ensure that the elderly people recalled food items consumed in the past given their age, food models, pictures and kitchen utensils were used. Only elderly people who signed the written informed consent form were included in the current study. Elderly people who were unable to recall what they ate were excluded from the study. An expert from the Department of Linguistics translated the questionnaire into the local languages used in Vhembe district, which are Tshivenda and Xitsonga, then back to English to ensure accuracy of the translation. The questionnaire was tested in a pilot study to determine whether the questions were clearly understood by the participants. To check for reliability, 10% of the questionnaire was randomly selected and elderly people were interviewed again one week after the initial interview by a different fieldworker. The quality control data were analysed in the same way as the original data for comparison.

The International Society for the Advancement of Kinanthropometry’s guidelines for doing anthropometric evaluations were followed. The adults were dressed in light clothing and barefoot, and the following measurements (standing height and weight) were made twice with calibrated equipment.^19^ A portable height gauge was used to measure height to the nearest 0.1 cm (Seca model 213, Hammer Steindamm, Hamburg, Germany), and weight was measured to the nearest 0.01 kg on a portable Seca solar scale (Seca model 0213, Hammer Steindamm, Hamburg, Germany). The solar scale and portable height measures were calibrated before measurements using a known weight and steel tape, respectively.

### Food survey

The quantitative food frequency questionnaire was used to assess the dietary intake and dietary patterns of elderly people. The adequacy of nutrient intake was compared with the recommended dietary intake for elderly people.^20^

### Definitions of underweight, normal, overweight and obesity

The body mass index (BMI) (kg/m^2^) was selected to estimate the prevalence of underweight, overweight and obesity according to the World Health Organization (WHO) reference values. Underweight was defined as < 18.5, normal weight as between 18.5 and 25.0, overweight as between 25.0 and 29.05 and obesity as > 30 kg/m^2^.^21^

### Ethical considerations

The study was approved by the Ethics Committee of the University of Venda and an ethical clearance certificate was issued (reference number SHS/19/NUT/12/2405), and permission to conduct the study was granted by the chiefs of the villages. The Declaration of Helsinki,^22^ good clinical practices and South African law were all followed in the conduct of the study. The participants received a thorough and adequate oral and written description of the study.^23^ Participants gave written signed informed consent to participate in the study. The consent form included the participants’ right to withdraw from the study, and codes were used to ensure confidentiality of the information obtained. In cases where the BMI or eating habits were classified as abnormal, fieldworkers gave nutrition counselling and referred the participants to professional nutritionists and dieticians.

### Data analysis

The Statistical Package for the Social Sciences (SPSS) version 25 (IBM Corporation, Armonk, New York, United States) was used to analyse categorical and descriptive data such as mean, standard deviation (s.d.), frequencies and percentages. The chi-square test for categorical data and Pearson’s correlation were performed to assess the relationship between sociodemographic characteristics and nutritional status. A *p* ≤ 0.05 was considered statistically significant. FoodFinder (Medical Research Council of South Africa, Tygerberg, South Africa) was used to analyse the dietary intake of the study participants. The dietary diversity score was interpreted using the WHO recommended cut-off point.

## Results

The sociodemographic information of the study participants is summarised in Table 1. Half of the study participants had ages within 60–69 years. The mean age (s.d.) of the male participants was 70.04 (6.65), while that of the female participants was 68.77 (7.35). The majority of the participants had attended school. Of all those who had attended school, 38.6% attended primary, while 28.1% attended secondary school. Half of the participants were married. Almost all participants were receiving social grants. Most of the participants were Venda speaking while a minority were Sepedi speaking.

**TABLE 1:** Sociodemographic characteristics of study participants (*N =* 300).

Variable	Gender				Total number of participants	
	Male		Female			
	*n*	%	*n*	%	*n*	%
**Age categories (in years)**						
60–69	70	23.0	105	35.0	175	58.0
70–79	47	16.0	44	15.0	91	31.0
80 and above	15	5.0	19	6.0	34	11.0
**Educational level**						
Primary school	55	18.3	61	20.3	116	38.6
Secondary school	34	12.0	50	17.0	84	28.1
Never attended school	43	14.0	57	19.0	100	33.3
**Social grant**						
Receiving	128	42.6	158	52.4	286	95.0
Not receiving	4	1.0	10	3.0	14	5.0
**Other source of income**						
Yes	34	11.0	58	19.0	92	30.0
No	99	33.0	111	37.0	208	70.0
**Marital status**						
Single	9	3.0	7	2.0	16	5.0
Married	103	34.0	65	22.0	168	56.0
Divorced	4	1.0	2	1.0	6	2.0
Widowed	18	6.0	93	31.0	111	37.0
**Ethnic group**						
Venda-speaking	132	44.0	162	54.0	294	98.0
Tsonga-speaking	0	0.0	6	2.0	6	2.0
**Religion**						
Christianity	88	29.0	134	45.0	222	74.0
Never attend church	45	15.0	33	11.0	78	26.0

Note: Age category: Male - mean = 70.04, ± s.d. = 6.65; Female - mean = 68.77, ± s.d. = 7.35.

s.d., standard deviation.

Table 2 provides the association between the sociodemographic and nutritional status of the study participants. The prevalence of underweight, overweight and obesity was 2.0%, 34.0% and 16.7%, respectively. The prevalence of overweight and obesity was high in women (18.7% and 11.7%) as compared to their male counterparts (15.3% and 5.0%). This finding was statistically significant (*p* < 0.05).

**TABLE 2:** Association between sociodemographic characteristics and nutritional status (*N* = 300).

Characteristics	Underweight		Normal		Overweight		Obese		*p*
	*n*	%	*n*	%	*n*	%	*n*	%	
**Gender**									
Male	5	1.7	66	22.0	46	15.3	15	5.0	0.043
Female	1	0.3	76	25.3	56	18.7	35	11.7	
**Age (years)**									
60–69	1	0.3	80	27.0	63	21.0	30	10.0	0.824
70–79	2	0.7	44	14.7	31	10.3	15	5.0	
80 and above	3	1.0	18	6.0	8	2.7	5	1.7	
**Educational level**									
Primary school	3	1.0	66	22.0	48	16.0	20	6.7	0.293
Secondary school	0	0.0	30	10.0	20	6.7	12	4.0	
Never attended school	3	1.0	46	15.3	34	11.3	18	6.0	
**Social grant**									
Receiving	6	2.0	141	47.0	97	32.3	42	14.0	0.000
Not receiving	0	0.0	1	0.3	5	1.7	8	2.7	
**Other source of income**									
Yes	3	1.0	34	11.3	31	10.3	24	8.0	0.011
No	3	1.0	108	36.0	71	23.7	26	8.7	
**Marital status**									
Single	0	0.0	10	3.3	6	2.0	1	0.3	0.073
Married	5	1.7	71	23.7	53	17.7	39	13.0	
Divorced	0	0.0	5	1.7	2	0.7	0	0.0	
Widowed	1	0.3	56	18.7	41	13.7	10	3.3	
**Ethnic group**									
Venda-speaking	6	2.0	142	47.3	100	33.4	46	15.3	0.033
Tsonga-speaking	0	0.0	0	0.0	2	0.7	4	1.3	
**Religion**									
Christianity	4	1.3	105	35.0	74	25.0	40	13.4	0.803
Never attend church	2	0.7	37	12.3	28	9.3	10	3.3	

Note: Pearson chi-square test. Significance at *p* < 0.05.

Table 3 provides the meal pattern of the study participants. Two-thirds of the participants (66.0%) ate three meals per day. Of all the participants, 86.3% of them ate breakfast every day, while 13.7% skipped breakfast. All participants ate lunch every day, and 66.7% ate supper every day. However, 19.6% ate supper sometimes while 13.7% did not eat supper at all. A majority of the participants (84.3%) did not eat snacks between meals. The minority of the participants drank alcohol (28.0%) and smoked (12.7%).

**TABLE 3:** Meal patterns of study participants.

Variables	*n*	Percentage (%)
**Number of main meals in a day**		
Once per day	0	0
Twice per day	82	27.3
Three per day	198	66
More than three times per day	20	6.7
**Breakfast skipping**		
Yes	41	13.7
No	259	86.3
**Breakfast**		
Everyday	259	86.3
Sometimes	0	0
Do not eat	41	13.7
**Lunch**		
Everyday	300	100
Sometimes	0	0
Do not eat	0	0
**Supper**		
Everyday	200	66.7
Sometimes	59	19.6
Do not eat	41	13.7
**Eats snacks**		
Yes	47	15.7
No	253	84.3
**Alcohol intake**		
Yes	84	28
No	216	72
**Tobacco use**		
Yes	38	12.7
No	262	87.3

All participants ate cereal (maize meal porridge, bread). A majority of the participants (70.3%) ate vitamin A–rich vegetables such as pumpkin, carrots, squash or sweet potatoes that are orange inside and tubers such as white potatoes, white yams, white cassava or other foods made from roots two to three times per week, while 29.7% ate vitamin A–rich vegetables and tubers 4–6 times per week. Moreover, 59.7% of the participants ate vitamin A–rich fruits 4–6 times per week, as compared to 40.3% of the participants who ate vitamin A-rich fruits 2–4 times per week. Almost all participants (96.3%) ate dark green leafy vegetables such as amaranth, cassava leaves, kale and spinach 4–6 times per week, while 3.7% ate dark green vegetables 2–3 times per week. However, 37.0% of participants ate flesh of animals such as beef, pork, lamb, goat, rabbit, game and chicken 2–3 times per week. More than half of the participants (56.0%) ate milk and dairy products 2–3 times per week (Table 4).

**TABLE 4:** Frequency of different food groups intake by study participants in a week.

Food groups	2–3 times per week		4–6 times per week	
	*n*	%	*n*	%
Cereals	0		300	100.0
Vitamin A–rich vegetables and tubers	211	70.3	89	29.7
White tubers and roots	58	19.3	242	80.7
Dark green leafy vegetables	11	3.7	289	96.3
Other vegetables (e.g. tomato, onion), including wild vegetables	8	2.7	292	97.3
Vitamin A–rich fruits	121	40.3	179	59.7
Other fruits (other fruits, including wild fruits)	214	71.3	86	28.7
Organ meat (iron-rich)	73	24.3	0	-
Flesh meats	111	37.0	0	-
Eggs	116	38.7	0	-
Fish	195	65.0	0	-
Legumes, nuts and seeds	216	72.0	0	-
Milk and milk products	102	34.0	0	-
Oils and fats	218	72.7	82	27.3
Sweets	0	-	0	-
Spices, condiments	86	28.7	0	-
Beverages	197	65.7	103	34.3

Table 5^10,48^ illustrates the daily nutrient intake of study participants. Half of the participants did meet their energy intake, while 49.8% did not meet their energy intake (*p* = 0.023). The mean intake of energy was 12345 kJ ± 3942.35 kJ. Of all the participants, 62.5% did not meet their protein intake, while 37.5% did meet their protein intake. The mean intake of protein was 45.3 g ± 19.4 g, while that of iron was 8.4 mg ± 7.5 mg and that of zinc was 11.2 mg ± 10.8 mg. Almost 60.0% of the participants did not meet their vitamin B2 intake, while only 40.2% did meet their vitamin B2 intake (Table 5).

**TABLE 5:** Mean daily nutrient intake of the participants (*n* = 300).

Nutrients (units)	Mean daily intake			Participants who did not meet reference intake		Participants who met or exceeded reference intake		*p*
	Mean ± s.d.	RDA	Range	*n*	%	*n*	%	
Energy (kcal)	12 345 ± 3942.35	12 881	1845–2328	149	49.8	151	50.3	0.358
Carbohydrates (g)	385 ± 127.34	130	93.4–230.4	80	26.7	200	73.3	0.451
Protein (g)	45.3 ± 19.4	56	41.3–78.2	187	62.5	112	37.5	0.046
Fats (g)	30.45 ± 27.6	ND	12.2–35.4	49	16.3	251	83.8	0.536
Calcium (mg)	845.23 ± 345.21	1000	120.6–984.5	169	56.4	131	43.6	0.023
Sodium (mg)	1.2 ± 0.94	1.3	0.45–1.18	97	32.3	203	67.8	0.674
Potassium (mg)	4.9 ± 4.6	4.7	0.32–4.89	218	72.7	82	27.3	0.523
Iron (mg)	8.4 ± 7.5	8	7.6–8.7	155	51.7	145	48.3	0.026
Zinc (mg)	11.2 ± 10.8	11	8.5–11.45	151	50.3	149	49.7	0.013
Folate (mg)	428.20 ± 415.23	400	389.5–486.7	130	43.3	170	56.8	0.034
Vitamin B1 (mg)	0.80 ± 0.57	1.2	0.8–1.29	185	38.4	115	38.4	0.672
Vitamin B12 (mcg)	0.84 ± 0.53	1.3	0.7–136	179	59.8	121	40.3	0.026
Vitamin B6 (mg)	0.43 ± 0.59	1.7	0.24–18.4	149	49.8	151	50.3	0.361
Vitamin C (mg)	93.5 ± 101.2	90	86.5–123.2	169	56.4	131	43.6	0.001
Vitamin A (mcg)	923 ± 245.3	900	784.6–255.4	64	21.3	236	78.8	0.052

*Source*: Please see the full reference list of the article, Nutrition Information Centre University of Stellenbosch (NICUS),^48^ for more information

ND, not determined.

## Discussion

The ageing process is a biological reality which has its own dynamics, largely beyond human control; like any other ageing people, the elderly in this study might have experienced these dynamics which are beyond their control.^24^ However, it is also dependent on how each society makes sense of old age. Most elderly people in the current study fell within the age range 60–69 years, and this reflects the picture of the elderly in South Africa; the life expectancy is 64 years and the *Older Persons Act* 13 of 2006 in South Africa provides that an older person is a person who is 60 years old or older.^25^ However, in South Africa, elderly people are defined as those between 50 and 65 years of age, depending on the setting, region and country.^5^ The process of ageing itself affects nutritional features. As people get older, they tend to eat less and make different food choices. These dietary changes might have a significant impact on the health status of these elderly people. Inadequate intake of food is directly linked to insufficient intake of necessary nutrients, which may pose a serious health problem and diet-related illnesses. The physiological changes that these elderly people experience at this age contribute to lower intake of food which had significant nutritional status.

The findings of the study showed low prevalence of underweight among elderly people, which was similar to previous studies conducted in South Africa^26^ and other parts of sub-Saharan countries.^2,12^ This study supports the findings of Mahan and Escott-Stump^27^ that the actual prevalence of underweight among older adults is quite low; these studies exhibit that being underweight is not a public health problem among elderly people. Improvement in the underweight of the elderly people in the current study is attributed to the fact that a vast majority of them had not skipped meals. Underweight among elderly people may be due to socio-economic factors, poor mobility and inability to purchase and prepare food.^28^

Our data found that 34.0% of elderly people were overweight, while 16.7% were obese. These findings are congruent with the previous study that indicated that KwaZulu-Natal in South Africa had the highest rate of overweight and obesity.^14,15^ The prevalence of overweight in the current study was slightly higher in elderly women (18.7%) when compared with 15.3% of elderly men. Furthermore, prevalence of obesity was higher in elderly women as compared to elderly men (11.7% vs 5.0%). These figures are still smaller than in younger people but have significant health implications considering the increased risk of comorbid conditions in the elderly. Similar findings were observed in sub-Saharan countries like South Africa, Ghana, Kenya, Senegal, Uganda, Botswana and Cameroon.^2,12,29^ The findings of this study were supported by Mahan and Escott-Stump,^27^ where prevalence of obesity has increased in all ages. Older adults are no exception; obesity rates are greater among those aged 65–74 years than among those aged 75 years and over, and obesity is associated with increase in mortality and contributes to many chronic diseases. Prevalence of obesity and overweight could be because ageing is associated with a decrease in total energy expenditure, and if this coincides with a maintained or increased energy intake, overweight or obesity may develop.^9^ However, in many rural communities, the socio-economic disparities and poor quality of traditional diets may contribute to malnutrition among elderly people. In the current study, undernutrition and overnutrition among the elderly co-exist, as in many sub-Saharan countries.^30,31^ The prevalence of obesity was high among women at 13.0%, as compared to their male counterparts at 4.0%.^32^ This may be because women are heavier than men while men are taller than women. Fat accumulation is lower in men and higher in women, due to essential body fat deposited in the mammary glands, and it can be further explained by differences in hormones, hormone receptors and enzyme concentrations.^19,33^ In many African countries, including South Africa, overweight and obesity are associated with a perception of ‘wealthy and healthy’, but this perception has drastically changed on the basis that overweight and obesity are associated with many noncommunicable diseases.^34^

Almost three-quarters of the elderly people were Christians. In South Africa, people tend to avoid certain foods because of their religious beliefs, and this is likely to affect their dietary intake and dietary practices. Some avoid certain foods because of personal dislikes, social and cultural practices and religion. However, the researchers did not ask about the cultural practices of the study participants, and this could have given a better insight into the dietary intakes. Every community has its own traditions, customs and beliefs around the types of foods that are healthy for people of different ages and those that are not. What is offered affects how individuals view food as well.^10,35^ Most of the elderly people know the nutritional values of the foods they eat. The dietary pattern of the participants shows that most of them ate three main meals a day, which is necessary for good health. This is consistent with research on elderly people conducted in rural South African countries.^36^

As shown by Shahar et al.,^36^ the older population tends to continue the more traditional eating pattern of three meals a day. However, a minority of them either skip breakfast or eat in between meals. A minority of the participants sometimes skip breakfast or dinner. Skipping of meals is a very common practice among elderly people in the rural areas.^36^ Skipping meals may aggravate dyspepsia and be consequential among elderly patients with diabetes on treatment. A minority of the participants in the current study snacked in between meals, possibly to enable them cope with the energy needs of the body as they go about their daily activities. However, Lee et al.^10^ reported low intake of nutrients among elderly people who snacked frequently. Insufficient intake of nutrients can cause weakened immune systems, which can increase the risk of infections, leading to weight loss and nutritional deficiencies.

The pattern also shows a low intake of alcohol and tobacco use among elderly people. Apart from the direct health-negative effects of alcohol and tobacco use, most elderly in rural areas are poor, and if they smoke or drink harmfully at the current price of cigarettes and alcohol, they may not be able to concurrently afford food rich in protein and micronutrients.^37^ Tachi et al.^38^ posit that elderly people are likely to adopt this simple and cost-effective measure, such as low intake of alcohol and increase of food intake, to reduce the risk of diseases and improve their health status.

The findings on food consumption revealed that many participants in the current study consumed food from different food groups. However, most of the participants consumed fruits, vegetables and cereals. Protein food sources were rarely consumed except that participants consumed legumes, nuts and seeds 2–3 times per week. These findings are congruent with previous studies that reported high intake of vegetables and cereals among older persons in South Africa^39^ and Zambia.^31^ In addition, maize meal porridge is one of the staple foods in the South African diet and is usually consumed with green leafy vegetables and a small amount of animal-derived food.^14^ The rare consumption of these animal-derived food sources could be because of their high cost, loss of teeth which makes it difficult for them to chew meat and, in some cases, religious or cultural practices. The prevalence of unhealthy eating is common among people in low socio-economic status.^40^ The high cost of food discourages consumption of food.^41^ The framework of public policies using subsidies and taxes could be implemented to manipulate the cost of food prices in order to make essential foods more affordable.^41^ Insufficient consumption of these animal food sources leads to low intake of important micronutrients among this age group.^42^

The findings of the study showed low consumption of milk and dairy products among the participants. Perhaps this can be attributed to the fact that milk is perceived as tasteless by most elderly people due to deterioration of taste buds.^27^ Between the ages of 75 and 85 years, there is a 65% reduction of the sensitivity of taste buds to sweet and salty tastes.^27^ The impressions of sensory stimuli related to food are one of the well-established elements that influence meal choice. The individual interprets the chemical and physical characteristics of food in terms of sensory qualities. These five sensory characteristics of food are categorised into five categories: appearance, taste, trigeminal mouth feel, and aroma.^30,42,43^ The ability to perceive these sensory attributes in addition to liking them determines whether an individual consumes a food or not.^25,44^ The low consumption of milk and dairy products can explain the reason why participants did not meet their dietary intake of calcium. Congruently, in Zambia, Maila et al.^31^ reported low consumption of milk and dairy products. However, in Kenya, Munoru^44^ reported high consumption of milk among elderly people. In Kenya, the role of milk in the traditional diet varies greatly between different tribes. In some tribes, milk from the livestock is the preferred staple food.^45^

About 38% of the study participants had the highest dietary diversity score. This may be because almost all participants received social grant. A social grant is given to qualifying poor households as an important contributor in reducing poverty and food insecurity in South Africa. Socio-economic status plays a vital role in quality of life, including the food choices that these elderly people can make.^13^ However, a study conducted by Chakona and Shackleton^46^ in South Africa revealed that grant recipients use the money not only on food but also on other household necessities. Alao et al.^30^ revealed that income remains a strong determinant in the choice of food consumed by elderly people. Furthermore, the highest dietary score may be linked to overnutrition observed among study participants, as indicated by the prevalence of overweight and obesity in the current study.

Almost half of the participants did not meet their energy intake in the current study. Alao et al.^30^ reported that elderly people may not eat enough to meet their energy needs, which can result in increases in the risk of nutrition-related illnesses. The present study revealed that although many participants consumed food from different food groups, they did not meet the dietary intake of iron, zinc, calcium and vitamins B1, B2 and C. These may be attributed to the age-related alterations to the sense of taste, which is closely linked to poor appetite, inappropriate food choices and lower nutrient intake.^27,47^ This insufficient intake of micronutrients may also be due to low intake of protein, as proteins share the same food sources with zinc, iron and vitamins B1 and B2.^27^

The limitation of the present study was that the information on dietary intake relied on the ability of the participants to recall, which might have resulted in under- and overreporting. However, the study may be underpowered to determine significant differences and associations because of reduction of the sample size from 400 to 300 as a result of participant withdrawals.

## Conclusion

This study demonstrated that most elderly persons had good nutritional status and dietary patterns. However, despite knowing the roles and importance of good nutrition, the inadequate intake of supplementary meals and micronutrients predispose this elderly population to malnutrition and micronutrient-related disorders. Understanding of dietary patterns of older people is necessary for providing appropriate nutritional guidance, and nutrition education to encourage healthy eating among elderly people should therefore be conducted frequently.
